# Evaluation of the Oncogenic Human Papillomavirus DNA Test with Liquid-Based Cytology in Primary Cervical Cancer Screening and the Importance of the ASC/SIL Ratio: A Belgian Study

**DOI:** 10.1155/2014/536495

**Published:** 2014-02-18

**Authors:** Xavier Catteau, Philippe Simon, Jean-Christophe Noël

**Affiliations:** ^1^Faculty of Medicine, Free University of Brussels, Brussels, Belgium; ^2^Department of Pathology, Institute of Pathology and Genetics, Avenue Georges Lemaître 25, 6041 Gosselies, Belgium; ^3^Unit of Gynecology, Erasme University Hospital-ULB, Brussels, Belgium; ^4^Unit of Gynecopathology, Department of Pathology, Erasme University Hospital-ULB, Brussels, Belgium

## Abstract

*Objectives*. In Belgium, very few studies have focused on cervical high-risk human papillomaviruses (hrHPV) prevalence and the relationship between HPV and cervical cytological abnormalities. The aim of this study was to investigate hrHPV prevalence and its relationship with cytological screening and histological results in the French-speaking community in Belgium (Brussels and Wallonia). *Methods*. A total of 58,265 liquid-based cytology tests were performed during this period. All cases of ASC-US, ASC-H, LSIL, and HSIL were tested by Hybrid Capture 2 for hrHPV screening. *Results*. The prevalence of cytological abnormalities was 3.1% for ASC-US, 0.3% for ASC-H, 1.5% for LSIL, and 0.3% for HSIL. The frequency of hrHPV infection was 47% in ASC-US, 90% in ASC-H, 86% in LSIL, and 98.4% in HSIL. CIN 2+ lesions were found in 12.2% of smears with an ASC-US result, in 54% of smears with an ASC-H result, in 12.5% of smears with a LSIL result, and in 89.3% of smears with a HSIL result. The ASC/SIL ratio was 1.9%. *Conclusions*. This study provides a good representation of cytological abnormalities and HPV status in patients living in Belgium's French-speaking community. The prevalence in our study was similar to that derived from meta-analyses of European studies. Our ASC/SIL ratio was 1.9%, being within the lower and upper limits proposed in the literature, which tends to prove the good quality diagnosis of cervical smears in our laboratory.

## 1. Introduction

Invasive cervical cancer (ICC) is the second most common female cancer worldwide and the third most common in Belgium [1, 2]. In Belgium, the incidence is 14.1 (Truncated age-standardised incidence rate using World Standard Population (TWSR) = 11.8) per 100,000 women per annum, while the mortality rate is 4.1 (TWSR = 2.6) per 100,000 women per annum [2]. The highest incidence of ICC in Belgium has been observed in the Brussels Capital Region. The mean age at diagnosis is 54 years. However, cervical cancer remains a rare cause of death in Belgium (1.6%) [2]. The identification of a strong causal relationship between persistent infection of the genital tract with oncogenic human papillomavirus (HPV) types and the occurrence of cervical cancer precursors and cervical cancer has resulted in the development of tests for HPV nucleic acid detection [3, 4]. ICC incidence and mortality rates have dramatically declined over the past five decades in developed countries, largely thanks to screening programs and HPV detection [5, 6]. In Belgium, very few studies have focused on cervical high-risk human papillomaviruses (hrHPV) prevalence and the relationship between HPV and cervical cytological abnormalities. The aim of this study was to investigate hrHPV prevalence and its relationship with cytological screening and histological results in our population. The following objectives were addressed: (a) to assess the prevalence of HPV infection in the study population stratified by cytological abnormalities; (b) to evaluate the cross-sectional association between HPV infection and histological findings; (c) to apply the ASC/SIL ratio to assess the quality of cytological diagnosis in our laboratory. To the best of our knowledge, no epidemiological study of this kind has ever been conducted in Belgium's French-speaking Community.

## 2. Materials and Methods

The study protocol received approval from Erasme Hospital's institutional ethics and research review boards. A computer search was carried out from the Department of Pathology's archives from January 2007 to January 2012 to identify all women with cervical cytology. A total of 58,265 liquid-based cytology (LBC) tests were performed during this period. All smears were carried out in the Erasme hospital by gynecologists on women living either in Wallonia or in Brussels. For cervical cytological testing, a clinician used an Ayres spatula to collect cells from the transformation zone and a cytobrush to collect cells from the endocervical canal. Both the spatula and brush were rinsed directly in vials that contained 20 mL of cytological fluid (PreservCyt; Cytyc Corp, Boxborough, MA) for processing and production of a thin-layer cytological slide (ThinPrep; Cytyc Corp). Of these, 3,090 cases were ASC-US, ASC-H, LSIL, or HSIL lesions. Patients atypical glandular cells and adenocarcinoma on Pap smear were not included in this study. A colposcopy was carried out as soon as the ASC-US, ASC-H, LSIL, and HSIL lesions were identified from the smears. A biopsy was carried out during colposcopy on fresh, clinically suspect areas, on leukoplakic areas (acetic acid-positive areas), and iodine-negative areas (after application of Lugol's iodine). 2011 International Federation of Cervical Pathology and Colposcopy Colposcopic Terminology of the Cervix (Rio De Janeiro, Brazil) was used to coloscopic classification. All ASC-US, ASC-H, LSIL, and HSIL smears and all biopsies were reviewed by two pathologists (XC, JCN). The reports were based on WHO criteria for classification of histological diagnosis of CIN. All ASC-US, ASC-H, LSIL, and HSIL cases were tested by Hybrid Capture 2 (Qiagen, Gaithersburg, Md) for research of hrHPV (hc2). Normal cytology cases were not tested by hc2. A quantity of 4 mL of samples in PreservCyt was processed in the sample conversion kit (Qiagen, Inc., Mississauga, Canada) and tested with hc2 according to the manufacturer's recommendations, using the specific HPV RNA probe cocktail for carcinogenic HR-HPV types 16, 18, 31, 33, 35, 39, 45, 51, 52, 56, 58, 59, and 68. Presence or absence of HPV DNA in the specimen was defined according to its strength in relative light units (RLU) compared to 1 pg/mL HPV16 DNA-positive control (CO). The sample was considered positive when the ratio of RLU/CO was ≥1. The RLU/CO ratios also provided an estimate of the amount of HPV DNA in the specimens (i.e., the viral load in the sample). The ASC/SIL ratio was obtained by dividing the sum of all ASC cases by the sum of all SIL cases. SIL for this purpose included low-grade SIL, high-grade SIL, and carcinoma. Atypical glandular cells and adenocarcinoma were not included.

## 3. Results

**Figure 1:**
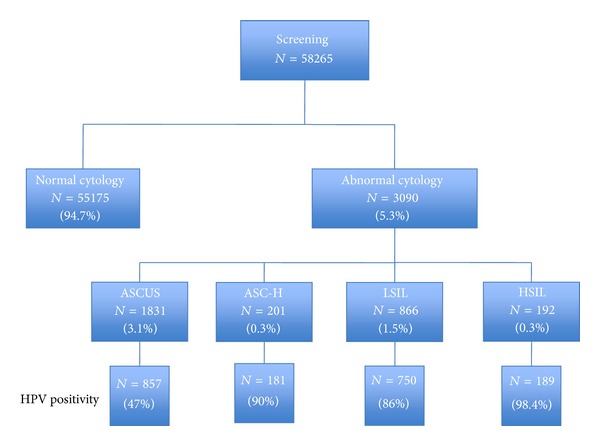
Distribution of cervical cytological abnormalities in a Belgian population.

The prevalence of cytological abnormalities in the population with microscopically interpretable samples was 3.1% for ASC-US, 0.3% for ASC-H, 1.5% for LSIL, and 0.3% for HSIL. The frequency of hrHPV infection was 47% in ASC-US, 90% in ASC-H, 86% in LSIL, and 98.4% in HSIL (Figure 1). Colposcopic biopsy results of 1,301 patients with pathologic Pap smear were obtained. Of 500 patients with ASC-US from Pap smear, 216 (43.2%) were negative (normal or metaplasia or atrophy or chronic cervicitis), 223 (44.6%) had CIN 1 and 61 (12.2%) had CIN 2+ (CIN 2+ CIN 3+ invasive carcinoma) upon biopsy. Of 185 patients with ASC-H from Pap smear, 32 (17.4%) were negative, 53 (28.6%) had CIN 1, and 100 (54%) had CIN 2+ upon biopsy. Of 448 patients with LSIL from Pap smear, 118 (26.4%) were negative, 274 (61.1%) had CIN 1, and 56 (12.5%) had CIN 2+ upon biopsy. Of 168 patients with HSIL on Pap smear, 11 (6.5%) were negative, 7 (4.2%) had CIN 1, and 150 (89.3%) had CIN 2+ upon biopsy (Figure 1).  Table 1 shows HPV test results stratified by cytology and histology results.

Overall, 89.8% of patients with CIN 1, 94.9% of patients with CIN 2, 100% of patients with CIN 3, and 100% of patients with invasive squamous cell carcinoma were hrHPV positive. As the main objective of screening was to highlight CIN 2+ lesions in the general population, we calculated the percentage of CIN 2+ lesions in relation to cytological lesions. In ASC-US, ASC-H, LSIL, and HSIL HPV positive lesions, the percentage of CIN 2+ was 15.4%, 55%, 18.2%, and 98.6%, respectively.

In ASC-US HPV negative lesions, the percentage of CIN 2+ was 2.4%. In HPV positive lesions, the percentage of CIN 2+ was 55%. Finally, the ASC/SIL ratio in our laboratory was 1.9%.

## 4. Discussion

We examined the prevalence of hrHPV infection in squamous intraepithelial lesions of the cervix in French-speaking Belgian women (Brussels and Wallonia), correlating it with cytological data. These data may assist the public health authorities in planning prophylactic and therapeutic strategies to prevent cervical cancer. One goal of the study was to assess the prevalence of HPV infection in our population, stratified by cytological abnormalities. While some of our patients were from Wallonia, the majority of patients were from the Brussels region, which also had the highest mortality rates of cervical cancer.

The cytological diagnosis of ASC-US is undesirable but unfortunately inevitable, and results from the morphological variability of squamous cells in different physiological and pathological conditions [7]. Although it is inevitable, the frequency with which the diagnosis of ASC-US is given should be minimized because its clinical monitoring remains controversial. Given the poor reproducibility of ASC-US diagnosis, colposcopy in these patients appears to be expensive and unnecessary and may lead to potential overtreatment [8, 9]. ASC-US lesions found by looking for hrHPV may at least be justified if one refers to the ASC-US/LSIL Triage Study (ALTS). In the aforementioned study, the authors demonstrated that the rate of ASC-US HPV positive lesions was 50.6% (47% in the present study) and that the ASC-US HPV positive lesions had an identical sensitivity to colposcopy for the diagnosis of CIN 2+ found in 11.4% of cases. In our study, the percentage of CIN 2+ lesions upon biopsy in ASC-US-HPV positive lesions was quite similar (15.4%). Also, as hrHPV positivity was found in 30–60% of ASC-US cases, many colposcopies proved unnecessary [10, 11]. Our results are similar to those in the literature where in ASC-US HPV positive cases, lesions are found to be between 5 and 18% of CIN 2+ lesions and 20 to 54% of CIN 1 lesions upon biopsy [8–10]. In our series, we found 2.4% of CIN 2+ who had an ASC-US-HPV negative smear. This rate is not negligible even with the conditions for monitoring gynecological smears in Belgium, whereby these patients will not benefit from smears within two years. For LSIL lesions, all studies agree that there is no reason to systematically investigate the presence of HPV. This is also the case in Belgium. Indeed, if we use strict cytological criteria, oncogenic HPVs are found in 70–90% of LSIL lesions [10, 12, 13]. In our population, 86% of LSIL lesions were hrHPV positive and we found 12.4% of CIN 2+ upon biopsy (Figure 1). Our study confirms the high hrHPV positivity rates in women with LSIL observed in the ASC-US/LSIL Triage study [14] and in meta-analyses [13, 15]. This data shows that the triaging capacity of a hrHPV cocktail test is quite low in the case of low-grade cytological abnormalities. However, the HPV test may be of interest in monitoring patients with a normal or satisfactory colposcopy and an LSIL-persistent smear. ASC-H lesions have rarely been studied in the literature [16, 17]. They represent only 0.3% of total cytology in our population and 90% of them are hrHPV positive (Figure 1). We found 55.2% of CIN 2+ through biopsy. The search for HPV does not seem useful in this type of condition, except in cases of normal colposcopy and if the HPV test is negative because there is a strong presumption of false positive cytology [8, 10, 16]. Our prevalence was also similar to that derived from meta-analyses of European studies. The pooled hrHPV positivity rates were 71.9% (95% CI, 62.8–80.9%) in LSIL or CIN 1 lesions [18] and 88.3% (95% CI, 85.8–90.8%) in cytological HSIL or high-grade CIN 2 [19, 20]. In fact, as in invasive cancers, nearly all HSIL cases were reported to be hrHPV positive [21] (Figure 1). We compared our data to European and American studies with a similar methodology, particularly using the hc2 test.  Table 2 summarizes the distribution of cytological abnormalities in the general population in European countries (France and Germany) as well as in the United States [1, 22–24]. The first noteworthy point is that, in the present study, the percentage of cytological abnormalities is lower than in the other studies (5.3%). We also note that the percentage of ASC-US is slightly higher in the US than in Europe. In our population, the percentage of LSIL and HSIL appears lower compared to other studies. The ASC-H percentage, found only in the study by Monsonego et al., is similar. The percentage of HPV positive cases in terms of histological lesions is notably similar. Finally, the percentage of CIN 2+ in ASCUS, ASC-H, LSIL, and HSIL HPV positive lesions is similar to that reported by Kitchener et al. [22].

*Significance of the ASC/SIL Ratio.* To avoid potential misuse of the ASC interpretation, it is recommended that the frequency of ASC interpretation should be no greater than 5% of the patient population [25]. In our study, the rate of ASC-US was 3.1%. The ASC/SIL ratio was introduced as a quality control measure that was less dependent on the patient population, because the ASC and SIL rates would both increase in a laboratory with more high-risk patients [26, 27]. The recommended ASC/SIL ratio should be ≤3 [28]. An ASC/SIL ratio >3 may suggest overutilization of the ASC interpretation by a particular laboratory or pathologist. It is unknown whether there is a lower limit to this ratio that corresponds to decreased sensitivity or an ideal ratio that maximizes both sensitivity and specificity; however, it was suggested that ASC/SIL ratios of less than 1.5 might be a useful flag for suboptimal screening sensitivity. Our ASC/SIL ratio was 1.9%, being within the lower and upper limits proposed in the literature. This ratio can also be useful in evaluating the performance of cytotechnologists. It is no surprise that cytotechnologists with lower ASC/SIL ratios were more specific in their diagnoses than those with higher ASC/SIL ratios. Renshaw et al. conclude as follows: “A laboratory depends on its cytotechnologists to maintain adequate sensitivity and on its cytopathologists to maintain adequate specificity” [29]. We agree with this concept.

## 5. Conclusion

This study gives a good representation of cervical abnormalities and HPV status of women living in Belgium's French-speaking community (Wallonia and Brussels). The results of our study are very similar to those of other studies of the same type, particularly in France, our neighboring country. It allows us to situate Belgium in the European Union with regard to the distribution of cervical lesions. We hope that it will also allow other laboratories, in Belgium and elsewhere, to compare their data with ours and provide useful information to public authorities regarding this disease.

## Figures and Tables

**Table 1:** HPV test results stratified by cytology and histology results.

Biopsy						
	Normal (%)		CIN 1 (%)		CIN 2+ (%)	
	HPV+	HPV−	HPV+	HPV−	HPV+	HPV−
ASC-US	133 (26.6)	83 (16.6)	184 (36.8)	39 (7.8)	58 (11.6)	3 (0.6)
ASC-H	28 (15.1)	4 (2.2)	53 (28.7)	0 (0)	100 (54)	0 (0)
LSIL	68 (15.2)	50 (11.2)	184 (41.1)	90 (20)	56 (12.5)	0 (0)
HSIL	10 (5.9)	1 (0.6)	6 (3.6)	1 (0.6)	150 (89.3)	0 (0)

**Table 2:** Comparison of European and American studies on the distribution of cytological abnormalities in a screening population.

	Normal cytology (%)	Abnormal cytology (%)	ASC-US (HPV positivity) (%)	ASC-H (HPV positivity) (%)	LSIL (HPV positivity) (%)	HSIL (HPV positivity) (%)
Kitchener et al. [22] (UK)	85.6	14.4	7.9 (NS)	NS	3.9 (NS)	2.2 (NS)
Baseman et al. [23] (USA)	81.4	18.6	9.7 (44)	NS	3.9 (84)	2.8 (95)
Dalstein et al. [24] (France)	84.5	14.5	1.7 (54)	NS	7 (70)	5.8 (91)
Monsonego et al. [1] (France)	90.4	9.6	2.9 (27)	0.4 (47)	5.1 (63)	1.1 (96)
Current study (Belgium)	94.7	5.3	3.1	0.3 (90)	1.5 (86)	0.3 (98)

NS: not specified.
